# Fibronectin- and Bioactive Glass-Modified Alginate Scaffolds Support Limited Primary Cell Proliferation In Vitro yet Demonstrate Effective Host Integration In Vivo

**DOI:** 10.3390/jfb16100386

**Published:** 2025-10-15

**Authors:** Benedetta Guagnini, Andrea Mazzoleni, Adrien Moya, Arnaud Scherberich, Barbara Medagli, Ivan Martin, Davide Porrelli, Manuele G. Muraro, Gianluca Turco

**Affiliations:** 1 Department of Medicine, Surgery and Health Sciences, University of Trieste, 34129 Trieste, Italy; benedetta.guagnini@phd.units.it (B.G.); bmedagli@units.it (B.M.); gturco@units.it (G.T.); 2 Department of Biomedicine, University of Basel and University Hospital of Basel, 4031 Basel, Switzerland; andrea.mazzoleni@usb.ch (A.M.); adrien.moya@unibas.ch (A.M.); arnaud.scherberich@usb.ch (A.S.); ivan.martin@usb.ch (I.M.); 3 Department of Biomedical Engineering, University of Basel, 4123 Allschwil, Switzerland; 4 Department of Life Sciences, University of Trieste, 34127 Trieste, Italy

**Keywords:** alginate, fibronectin, bioactive glass, hydroxyapatite, bone scaffolds, mesenchymal stromal cells

## Abstract

Alginate-hydroxyapatite (AL) scaffolds modified with fibronectin (FN) or bioactive glass (BGMS10) have recently been characterized for their physicochemical properties and proposed as promising candidates for bone regeneration. Here, we present their first systematic biological evaluation, focusing on adhesion, proliferation, osteogenic differentiation, and in vivo host response. We compared FN-, BG-, and unmodified AL scaffolds using an immortalized mesenchymal stromal cell line (M-SOD) and primary human bone marrow-derived (BM-MSCs) and adipose-derived stromal cells (ASCs). FN scaffolds enhanced initial adhesion across all cell types and supported proliferation in M-SODs, but primary BM-MSCs and ASCs showed minimal expansion, regardless of scaffold type. BG scaffolds promoted expression of late-stage osteogenic markers in BM-MSCs, consistent with their ion release profile, but had limited impact on ASCs. In vivo subcutaneous implantation of acellular scaffolds in nude mice revealed robust host cell infiltration and extracellular matrix deposition across all scaffold types, confirming biocompatibility and integration. However, vascularization remained limited and did not differ substantially between formulations. Together, these findings highlight a critical discrepancy between immortalized and primary stromal cell responses to scaffold cues, underscoring the choice of cell source when evaluating the biocompatibility of a novel scaffold. At the same time, the effective in vivo integration observed across scaffold types emphasizes the importance of host tissue responses for translational evaluation of functional biomaterials.

## 1. Introduction

Advanced biomaterials for bone regeneration are a major focus in tissue engineering, aiming to repair defects caused by trauma, congenital abnormalities, or degenerative diseases. An ideal scaffold must integrate seamlessly with the host tissue, mimic the bone’s natural microenvironment, and provide structural support while promoting cellular proliferation and guiding stem cell differentiation into osteoblasts to achieve complete bone regeneration [1,2].

Among the various biomaterials available, alginate (Alg), a biodegradable polysaccharide derived from brown seaweed, has been extensively utilized due to its biocompatibility, low toxicity, and gelation properties that support cell proliferation and nutrient diffusion [3]. However, its biological inertness, limited osteoconductivity, and poor mechanical strength present significant challenges, often necessitating modifications or the incorporation of bioactive components to enhance its functionality [4,5].

To address these limitations, hydroxyapatite (HAp), a mineral mimicking bone’s inorganic matrix, was integrated to improve the scaffold’s mechanical strength and also enhance osteoconductivity [6]. Subsequent investigations from our group and others have confirmed that Alg/HAp scaffolds significantly improve cellular interactions and mechanical properties [5,7,8]. Nonetheless, further optimization is required to fully exploit their osteogenic potential, as their composition, porosity, and bioactive signals critically influence differentiation and regeneration [9]. To enhance bioactivity, two additional formulations were developed: one functionalized with fibronectin to improve cellular adhesion and proliferation, and another incorporating bioactive glass (BGMS10) [10], to stimulate osteogenesis and angiogenesis [11,12]. These formulations demonstrated enhanced bioactivity, biocompatibility, and structural properties, making them promising for bone regeneration.

However, immortalized cell lines often do not accurately reflect the complex behavior of primary cells, which are more physiologically relevant but exhibit distinct responses to biomaterial cues [13]. Furthermore, in vitro scaffold testing does not always predict in vivo outcomes [14], where host tissue interactions, immune response, and vascularization play critical roles in scaffold performance.

A crucial challenge in scaffold-based tissue engineering is bridging the gap between promising in vitro data and effective in vivo performance. Primary bone marrow-derived mesenchymal stromal cells (BM-MSCs) and adipose-derived stromal cells (ASCs) are relevant candidates for bone regeneration due to their osteogenic potential and availability [15,16], respectively, but their interactions with these alginate-based scaffold compositions remain poorly understood. Additionally, while bioactive glass has been widely recognized for its osteoinductive properties [12], its efficacy in enhancing osteogenic differentiation in primary cell populations, particularly in comparison to other alginate-based formulations, remains to be fully characterized. Moreover, in vivo conditions such as scaffold degradation, host cell infiltration, and vascularization introduce additional complexities that impact long-term biomaterial performance.

Based on these considerations, we hypothesize that scaffold composition significantly influences primary cell adhesion, proliferation, and osteogenic differentiation, with bioactive glass providing an advantage in osteogenic commitment. Furthermore, we hypothesize that scaffold structure and composition will impact early-stage host tissue integration in vivo, with potential cellular infiltration and remodeling differences over time.

To test these hypotheses, we evaluated three scaffold formulations, Alg/HAp (AL), Fibronectin-Alg/HAp (FN), and BGMS10-Alg/HAp (BG), for their ability to support adhesion, proliferation, and osteogenic differentiation of primary BM-MSCs and ASCs. In parallel, we implanted acellular scaffolds subcutaneously in immunodeficient mice to gain preliminary insights into host-material interactions, cellular infiltration, and early extracellular matrix deposition.

This study compares the responses of primary BM-MSCs and ASCs to those of an immortalized mesenchymal cell line on functionalized AL scaffolds, evaluating both in vitro outcomes and in vivo integration. By combining primary cell-based assays with ectopic implantation, we identify scaffold-specific limitations in supporting proliferation and osteogenic differentiation, and observe how host interaction may compensate for these in vivo. This dual approach addresses a persistent gap in scaffold evaluation pipelines, where biologically relevant cell models and in vivo integration studies are rarely combined.

## 2. Material and Methods

### 2.1. Culture Media

Three culture media formulations were used in this study: complete medium (CM), proliferation medium (PM), and osteoblastic differentiation medium (OB). The CM was composed of α-Modified Eagle’s Medium (α-MEM) supplemented with 10% fetal bovine serum (FBS), 2 mM GlutaMAX, 100 mM HEPES, 1 mM sodium pyruvate, 100 U/mL penicillin, 100 µg/mL streptomycin, and 5 ng/mL fibroblast growth factor-2 (FGF2). The PM (DAF medium) was prepared by supplementing CM with 10 nM dexamethasone, 0.1 mM ascorbic acid, and 5 ng/mL FGF2. The OB medium was obtained by enriching CM with 1 µM dexamethasone, 0.1 ascorbic acid, and 10 mM beta-glycerophosphate. Table S1 provides full details on all reagents used in this study, including catalog numbers.

### 2.2. Cell Source and Expansion

Bone marrow (BM) aspirates were obtained during routine orthopedic surgical procedures involving iliac crest exposure, while human adipose tissue samples were collected from patients undergoing liposuction or abdominoplasty. Informed consent was obtained under the general consent (Forschungskonsent) of the University Hospital Basel, approved by Swissethics (Art. 29, 32 Human Research Ordinance, HFV).

Freshly isolated BM nucleated cells were plated at 1 × 10^5^ cells/cm^2^ in CM, with medium changes twice per week. Upon confluence, BM-derived mesenchymal stromal cells (hBM-MSCs) were expanded at 3–5 × 10^3^ cells/cm^2^. Cells at passage 2 or 3 (p2–p3) were enzymatically detached using 0.05% trypsin/0.01% EDTA, counted, and used for 3D culture. Immortalized M-SOD (Mesenchymal Stromal Cells Sword of Damocles) cells were expanded in CM before seeding at p6, following Bourgine et al. [17].

Stromal vascular fraction (SVF) cells were isolated from adipose tissue via enzymatic digestion [16]. The resulting cells were seeded at 2 × 10^4^ cells/cm^2^ and expanded for one week. The adherent population, adipose-derived stromal cells (ASCs) p0, was detached with 0.05% trypsin/0.01% EDTA, reseeded at 3 × 10^3^ cells/cm^2^, and further expanded to p1. A total of 5 × 10^5^ ASCs at p1 were used for experiments [18,19].

### 2.3. Materials and Scaffold Manufacturing

Sodium alginate (MW = 135,000, FG = 0.67; FGG = 0.59), derived from *Laminaria hyperborea*, was obtained from FMC Biopolymers (Drammen, Norway). Hydroxyapatite (HAp), bioactive glass (BGMS10), glucono-delta-lactone (GDL), and fibronectin (1 mg/mL) were purchased from Merck KGaA (Darmstadt, Germany). Alginate/Hydroxyapatite scaffolds (AL) were fabricated following the protocol of Turco et al. [6], with BGMS10 bioactive glass incorporation (BG) and fibronectin functionalization (FN) prepared as described by Guagnini et al. [12] and Zumbo et al. [11], respectively. Briefly, a 2% (*w*/*v*) alginate and 3% (*w*/*v*) HAp suspension was mixed with 60 mM GDL, cast into molds, and left to gel overnight. Scaffolds were cryopreserved, freeze-dried, and sectioned into uniform 10 mm × 3 mm cylinders using a 3D-printed polylactic acid (PLA) slicer (Figure S1). For in vivo implantation, scaffolds were trimmed to 4 mm × 3 mm. Sterilization was performed via UV light (90 min), then deionized water washing and antibiotic treatment. Before use, AL and BG scaffolds were equilibrated in culture media for 24 h, while FN-coated scaffolds were pre-incubated in 10 µg/mL fibronectin solution for 24 h before equilibration.

The physicochemical and mechanical properties of these scaffolds have been extensively characterized in the cited studies, using identical raw materials and fabrication protocols. AL scaffolds exhibit >88% interconnected porosity with pores of 100–300 µm, a dry compressive modulus of ~6.3 MPa and ultimate strength of ~0.29 MPa, and a hydrated modulus of ~83 kPa. Incorporation of BGMS10 slightly reduces porosity (80.2 ± 1.1% at 0.3% *w*/*v* BG; 70.2 ± 0.6% at 0.6% *w*/*v* BG) and maintains comparable dry-state mechanical properties, while contributing ion release (Si, Ca, Na, P, Mg, Sr) known to stimulate osteogenesis. Fibronectin functionalization forms a thin, stable surface coating confirmed by μBCA assay, enhancing cell adhesion without altering scaffold morphology. Key parameters from the original characterization are summarized in Supplementary Table S2.

### 2.4. Adhesion and Proliferation

Scaffolds were tap-dried on sterile gauze for 5 s, transferred to a 24-well plate, and seeded with 1 × 10^6^ cells in 100 µL of complete medium (CM). After incubation at 37 °C in a humidified 5% CO_2_ atmosphere for 3 h to facilitate attachment, 1.5 mL of CM was carefully added per well, and scaffolds were maintained with medium changes every 3 days. Seeding efficiency and proliferation were assessed at 24 h and 7 days post-seeding using MTT and CellTiter-Glo assays. Seeding efficiency was calculated by comparing scaffold-attached cells at 24 h to 2D controls (100% retention). The proliferation rate was determined by comparing the 7-day signal to the 24 h 2D culture reference, indicating net cell expansion. For MTT assays, scaffolds were incubated with 700 µL of MTT solution (1 mg/mL) for 3 h at 37 °C in the dark. Formazan crystal formation was observed under an EVOS XL Core microscope to assess cell distribution, followed by solubilization in dimethyl sulfoxide (DMSO) and absorbance measurement at 560 nm. Cell-free scaffolds served as blanks. Additionally, the CellTiter-Glo Luminescent Assay was performed following the manufacturer’s protocol to quantify ATP levels, with results in the supplementary data.

### 2.5. Scanning Electron Microscopy (SEM)

Scanning Electron Microscopy (SEM) was used to analyze the surface morphology and microstructure of the scaffolds. After culture, samples were fixed overnight at 4 °C in 0.1 M sodium cacodylate buffer with 2% glutaraldehyde, followed by dehydration through a graded ethanol series (30–100%; 15 min per step) and critical point drying (Autosamdri-815, Tousimis, Rockville, MD, USA). Dried samples were mounted on aluminum stubs, sputter-coated with a 20 nm gold layer (LEICA EM Ace 600, Vienna, Austria), and imaged using a ZEISS Gemini 2 scanning electron microscope (Carl Zeiss Microscopy GmbH, Jena, Germany) at 5 kV primary electron beam voltage and 200 pA probe current, with magnifications ranging from 50× to 5000×. Longitudinal and transversal sections were qualitatively assessed for microstructural features.

### 2.6. RNA Extraction and Quantitative Real-Time PCR

Total cellular RNA was extracted using TRI Reagent solution combined with the Qiagen Miniprep Kit (QIAGEN GmbH, Hilden, Germany), following mechanical homogenization with the FastPrep-24 lysis system and stainless steel beads. RNA concentration and purity were assessed using a NanoDrop ND-1000 spectrophotometer (Thermo Fisher Scientific, Wilmington, DE, USA). For cDNA synthesis, 1 µg of RNA was reverse-transcribed using SuperScript III reverse transcriptase (Thermo Fisher Scientific, Waltham, MA, USA), following the manufacturer’s protocol. Quantitative real-time PCR (qRT-PCR) was performed using the ViiA 7 Real-Time PCR System with TaqMan Gene Expression Assays (both Thermo Fisher Scientific, Waltham, MA, USA). Gene expression levels for Runt-related transcription factor 2 (RUNX2), Osteocalcin (BGLAP), Ki-67, Osteopontin (OPN), Bone Sialoprotein (BSP), Collagen type I (COL1A1), and Alkaline Phosphatase (ALP) were normalized to GAPDH and analyzed using the 2^−ΔΔCT^ method to calculate fold changes relative to the control condition.

### 2.7. Whole Mount Immunostaining and Confocal Imaging

Tissue constructs were fixed overnight in 4% PFA at 4 °C, rinsed in PBS, and permeabilized with 0.4% Triton X-100 in PBS (PBS-T) for 10 min. Non-specific binding was blocked with 5% goat serum in PBS-T for 1 h. For DAPI-Phalloidin-Ki67 staining, constructs were incubated overnight at 4 °C in a blocking buffer containing FITC-Phalloidin and rat anti-human Ki-67 primary antibody (1:500). After PBS washes, samples were incubated for 4 h at room temperature with goat anti-rat Alexa Fluor 647 secondary antibody (1:1000). Nuclei were counterstained with DAPI (1:1000) for 10 min. Samples were mounted onto multiwell imaging slides and imaged using a Nikon AXR confocal microscope (Nikon Corporation, Tokyo, Japan).

### 2.8. In Vivo Ectopic Implantation in Nude Mice

Given the limited adherence and proliferation of primary cells observed in vitro, we elected to perform acellular subcutaneous implantation to evaluate scaffold colonization by host-derived cells and early extracellular matrix deposition. This model allowed us to isolate host–material interactions, independent of donor cell viability, and to adhere to the 3R principle by avoiding more invasive orthotopic models at this exploratory stage. The results were intended to provide a first validation of scaffold compatibility before advancing to critical-size bone defect models. Acellular scaffolds were subcutaneously implanted in Rj:NMRI-Foxn1<nu/nu> female nude mice (Janvier Labs, Le Genest-Saint-Isle, France). All animal procedures were conducted under the approval of the Cantonal Veterinary Office of Basel-Stadt (permit number BS 1797, and National no. 37404), in accordance with the Swiss Animal Welfare Act (TSchG, SR 455) and Ordinance (TSchV, SR 455.1), and in compliance with the 3Rs principles. The implantation protocol followed the procedure described by Osinga and colleagues [20].

### 2.9. Histological Processing and Staining

After differentiation or explantation, scaffolds were rinsed in PBS and fixed overnight in 4% paraformaldehyde (PFA) at 4 °C. For structural support, samples were embedded in HistoGel before paraffin embedding. Using a Thermo Scientific HM 355S microtome (Thermo Fisher Scientific, Waltham, MA, USA), 10 μm thick sections were cut and mounted onto SuperFrost Plus slides. Sections were deparaffinized, rehydrated, and stained to assess tissue morphology, mineralization, and extracellular matrix composition.

For Hematoxylin and Eosin (H&E) staining, sections were stained with hematoxylin (nuclei, blue) and eosin (cytoplasm, pink) to evaluate general morphology. Calcium deposition was assessed via Alizarin Red staining, where rehydrated sections were incubated in 1% Alizarin Red solution (pH 4.2) for 30 s and then thoroughly rinsed in distilled water. Mineralized regions appeared red-orange under light microscopy. Collagen deposition and tissue organization were analyzed using Masson’s Trichrome staining, where collagen fibers stained blue, cytoplasm/muscle fibers red, and nuclei dark brown/black. Stained sections were imaged using a Hamamatsu Nanozoomer S60 slide scanner (Hamamatsu Photonics K.K., Hamamatsu City, Japan).

### 2.10. Immunofluorescence Staining

Scaffold sections were deparaffinized in Ultraclear (3 × 15 min), rehydrated through a descending ethanol series, and rinsed in distilled water. Antigen retrieval was performed in a citrate buffer (pH 6) at 96 °C for 15 min, then cooling and PBS washes. Sections were blocked for 30 min in 5% animal serum in PBS-T (0.2%) to prevent non-specific binding.

Primary antibodies, rat anti-mouse CD31 (1:200) and rabbit anti-human Vimentin (1:200), were diluted in 1% serum in PBS-T and incubated overnight at 4 °C. After PBS-T and PBS washes, secondary antibodies, AlexaFluor 647 anti-Rat (1:400) and AlexaFluor 546 anti-Rabbit (1:400), were applied for 30 min at room temperature. Nuclei were counterstained with DAPI (1:500) for 10 min, and sections were mounted in Fluoromount Aqueous Mounting Medium before drying in the dark.

Immunofluorescence images were captured using a Nikon Ti2 widefield microscope equipped with a Nikon DS-Ri2 camera, CFI Plan Apo Lambda NA 0.75×, 20× objective, and NIS-Elements AR 5.21.03 software (all Nikon Corporation, Tokyo, Japan). Quantification of marker expression and nuclear content was performed using QuPath (v0.6.0) with the StarDist plugin for cell segmentation and classification.

### 2.11. Statistical Analysis

Statistical analyses were performed using GraphPad software (version 8.0.2). Data normality was assessed using the Shapiro–Wilk test. For normally distributed datasets, one-way ANOVA with Bonferroni’s correction was applied. Kruskal–Wallis and Mann–Whitney U tests were used for non-normally distributed data, both with Bonferroni’s correction. A *p*-value of α = 0.05 was considered statistically significant. For comparisons between two groups with unequal variances, Welch’s *t*-test was used instead of a standard *t*-test, as it does not assume equal variances and is more robust in cases of variance heterogeneity.

## 3. Results

**Figure 1:**
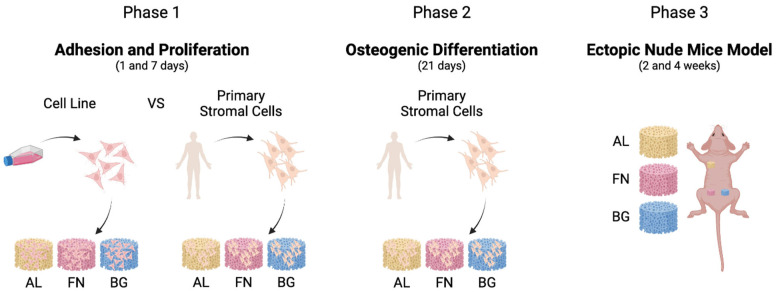
Schematic representation of the Study Design. AL: Alginate/Hydroxyapatite, FN, Alginate/Hydroxyapatite-Fibronectin, BG: Alginate/Hydroxyapatite-BGMS10. Cell Line: MSOD, Primary Stromal Cells: BM-MSCs, and ASCs. Created in BioRender. Martin, I. (2025) https://BioRender.com/hfr1iqk (accessed on 1 April 2025).

A sequential approach was employed to evaluate the performance of alginate-based composite scaffolds, as outlined in Figure 1. We first assessed cell adhesion and proliferation using M-SOD and primary mesenchymal stromal cells in vitro, followed by osteogenic differentiation studies. Finally, scaffold integration was examined in vivo using a subcutaneous ectopic model in mice.

Cell adhesion and proliferation were first assessed in vitro, revealing scaffold-dependent differences in stromal cell interaction. These were followed by osteogenic differentiation studies and in vivo implantation to evaluate tissue integration.

### 3.1. Fibronectin Promotes BM-MSC and ASC Adhesion and Growth, While Bioactive Glass Has Minimal Impact

**Figure 2:**
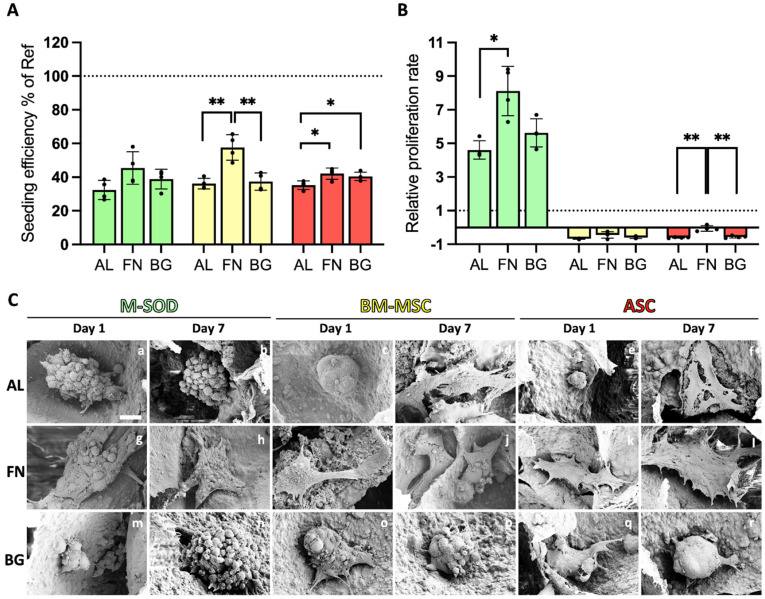
Seeding efficiency, proliferation, and cell morphology of cells on scaffolds. M-SOD (green), BM-MSCs (yellow), and ASCs (red) on AL, FN, and BG scaffolds, assessed via MTT assays and SEM. (**A**) Seeding efficiency at Day 1, expressed relative to 2D well-plate controls (dashed line at 100%). (**B**) Relative proliferation rate from Day 1 to Day 7, normalized to Day 1 values (dashed line at 1 on the Y-axis). Error bars represent standard deviation from quadruplicate measurements. Statistically significant differences are indicated (*: *p* < 0.05; **: *p* < 0.01). (**C**) SEM images of M-SOD, BM-MSCs, and ASCs on AL, FN, and BG scaffolds on Day 1 and Day 7, illustrating cell morphology and scaffold interaction. Scale bars = 10 µm.

The adhesion and proliferation of stromal cells on three scaffold formulations—Alg/HAp (AL), fibronectin-functionalized (FN), and BGMS10-incorporated (BG)—were assessed using MTT assays and SEM imaging (Figure 2). Bone marrow-derived mesenchymal stromal cells (BM-MSCs), adipose-derived stromal cells (ASCs), and immortalized M-SOD cells exhibited distinct interactions with the scaffolds.

FN-functionalized scaffolds significantly improved seeding efficiency for all cell types on Day 1 compared to AL scaffolds (Figure 2A), highlighting the role of FN in promoting initial cell adhesion, particularly for BM-MSCs. In contrast, BG scaffolds did not significantly enhance seeding efficiency over AL scaffolds, suggesting that bioactive glass alone does not provide additional adhesion cues for primary cells. The observed adhesion trends were further examined using MTT staining (Supplementary Figure S2), which showed higher signal intensity in FN scaffolds, potentially reflecting more significant metabolic activity in viable cell clusters. While all scaffolds demonstrated biocompatibility, ASCs exhibited greater MTT conversion, suggesting higher metabolic activity. However, since MTT only reflects metabolic function, this does not necessarily indicate improved adhesion efficiency or long-term proliferation.

Proliferation rates varied across different cell types and scaffold formulations. Immortalized M-SOD cells exhibited significantly higher proliferation than primary BM-MSCs and ASCs on all scaffolds from Day 1 to Day 7 (Figure 2B), consistent with previous studies on MG-63 osteosarcoma cells [11,12]. This highlights the inherently greater proliferative capacity of immortalized cell lines in scaffold studies. In contrast, primary BM-MSCs and ASCs showed minimal proliferation across all conditions, underscoring the challenges of sustaining primary cell expansion on these formulations. FN scaffolds slightly improved metabolic activity and retention for primary cells, but this did not translate into a significant proliferation increase. These findings reinforce the role of fibronectin in supporting initial cell attachment, though additional strategies may be required to enhance long-term primary cell growth.

SEM imaging (Figure 2C) provided critical insights into cell morphology and scaffold interactions. On AL scaffolds, cells predominantly exhibited a spherical morphology with limited adhesion, reinforcing alginate’s poor intrinsic bioactivity. In contrast, FN scaffolds supported well-spread and flattened cells, particularly ASCs, indicating strong adhesion and engagement with the scaffold surface. BG scaffolds presented a mix of rounded and partially spread cells, suggesting moderate adhesion, though not to the extent observed in FN scaffolds. Notably, some primary cells on AL scaffolds (Figure 2C(d–f)) displayed signs of cellular stress, including membrane rupture (red arrows), highlighting the limitations of alginate as a biologically inert material.

Together, these results show that FN functionalization significantly improves cell adhesion and retention, while BG alone does not enhance early cell-scaffold interactions. These findings highlight the importance of scaffold bioactivity in promoting initial cell attachment, especially for primary stromal cells, though additional strategies may be required to support long-term proliferation.

### 3.2. Scaffold Composition Influences Osteogenic Differentiation: BG Supports BM-MSCs, ASCs Remain Undifferentiated

**Figure 3:**
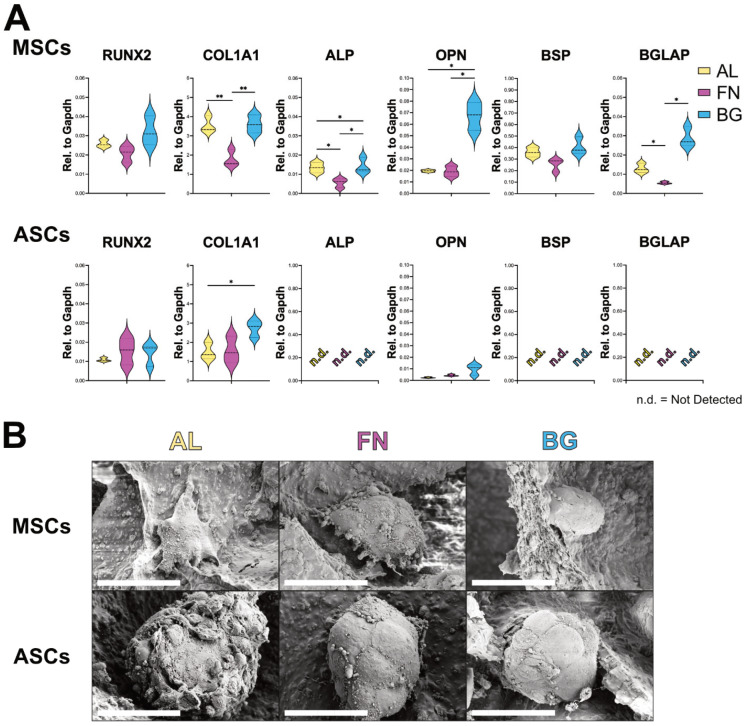
Osteogenic differentiation of BM-MSCs and ASCs on AL, FN, and BG scaffolds. (**A**) Gene expression levels of RUNX2, COL1A1, ALP, OPN, BSP, and BGLAP in BM-MSCs (top) and ASCs (bottom) after 21 days in osteogenic media. Results represent mean ± SD from three scaffolds per formulation. n.d. = not detected. Statistically significant differences are indicated (*: *p* < 0.05; **: *p* < 0.01). (**B**) SEM images of BM-MSCs and ASCs on AL, FN, and BG scaffolds after 21 days in osteogenic media. High-magnification SEM images show the compact, spherical morphology of cell clusters interacting with the scaffold surface. Scale bars = 50 µm.

Following the assessment of adhesion and proliferation, we evaluated the osteogenic differentiation potential of BM-MSCs and ASCs cultured on AL, FN, and BG scaffolds. Gene expression analysis of key osteoblastic markers (RUNX2, COL1A1, ALP, OPN, BSP, BGLAP) was performed via RT-qPCR after 21 days in osteogenic conditions to assess the impact of scaffold composition on differentiation (Figure 3A). BM-MSCs (top row) exhibited scaffold-dependent differentiation patterns. While RUNX2 expression remained unchanged across conditions, COL1A1 expression was significantly higher on AL and BG scaffolds, suggesting that these formulations promote extracellular matrix deposition. This could be attributed to the stiffer nature of AL and BG scaffolds, which may provide mechanical cues for matrix synthesis. Notably, late-stage osteogenic markers (ALP, OPN, BSP, BGLAP) were significantly upregulated in BM-MSCs cultured on BG scaffolds, indicating a more advanced osteogenic maturation. This suggests that BG scaffolds enhance osteogenic differentiation, likely due to bioactive ion release that promotes cellular commitment to the osteoblastic lineage. In contrast, ASCs (bottom row) displayed minimal osteogenic differentiation across all scaffold types. While COL1A1 expression was elevated on AL and BG, late-stage osteogenic markers (BSP, BGLAP, ALP) were undetectable, suggesting that ASCs lack the intrinsic cues for osteogenic commitment.

SEM analysis (Figure 3B) revealed compact, rounded cell clusters in BM-MSCs and ASCs across all scaffold types, with no substantial morphological differences. This suggests that, despite variations in gene expression, cellular organization remains essentially unchanged.

These findings reinforce that scaffold composition plays a key role in osteogenic differentiation. BG scaffolds support BM-MSC maturation, while ASCs require additional cues to fully commit to the osteogenic lineage.

### 3.3. Scaffold Stability and Cell Morphology in Long-Term Culture

**Figure 4:**
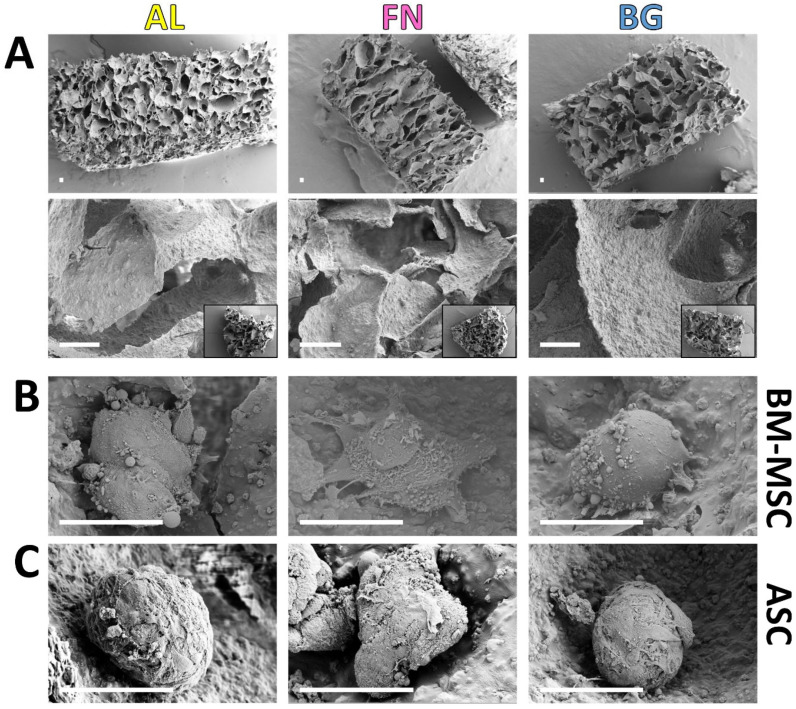
Scaffold Structural Integrity and Cell Morphology on Alginate-Based Scaffolds After Long-Term Culture. ((**A**), top row): SEM images of AL, FN, and BG scaffold cross-sections after 28 days in culture show preserved porous architecture with no visible signs of degradation or collapse. High-magnification SEM images ((**A**), bottom row) of scaffolds highlight the surface texture and pore structure. Insets show the entire scaffold structure at a lower magnification. Scale bars: 100 µm. BM-MSCs (**B**) and ASCs (**C**) interacting AL, FN, BG scaffolds, confirming the compact, round morphology of cell clusters and their interaction with the scaffold surface. Scale bars: 50 µm.

In addition to assessing cell morphology, we also examined the long-term structural stability of the scaffolds. Assessing scaffold structural integrity over time is crucial before in vivo implantation, as material degradation can affect tissue integration. To evaluate this, we analyzed the microstructural stability of AL, FN, and BG scaffolds after 28 days in culture using SEM imaging (Figure 4). The SEM images reveal that the inner scaffold network (top row) maintained its interconnected porosity, indicating sustained structural integrity under osteogenic conditions. Additionally, higher-magnification images (bottom row) highlight that surface texture and pore structure remained unchanged, reinforcing the stability of AL, FN, and BG scaffolds. These findings are consistent with previous studies [8,11], which reported scaffold stability for up to 14 days under different culture conditions. Our results extend this observation, demonstrating that these scaffold formulations can maintain their architecture for at least 28 days in culture, further supporting their potential for long-term bone tissue engineering applications. Beyond scaffold integrity, cellular morphology was examined in greater detail to further assess how different scaffold formulations influence cell behavior over prolonged culture periods (Figure 4B,C). Consistent with previous observations, BM-MSCs and ASCs exhibited similar morphologies across all scaffold formulations, predominantly forming compact spheroid-like clusters. These findings reinforce the earlier structural analysis, confirming that scaffold composition does not significantly impact long-term cell morphology.

### 3.4. Alginate-Based Scaffolds Support Early Cell Infiltration and Collagen Deposition, but Lack Vascularization

**Figure 5:**
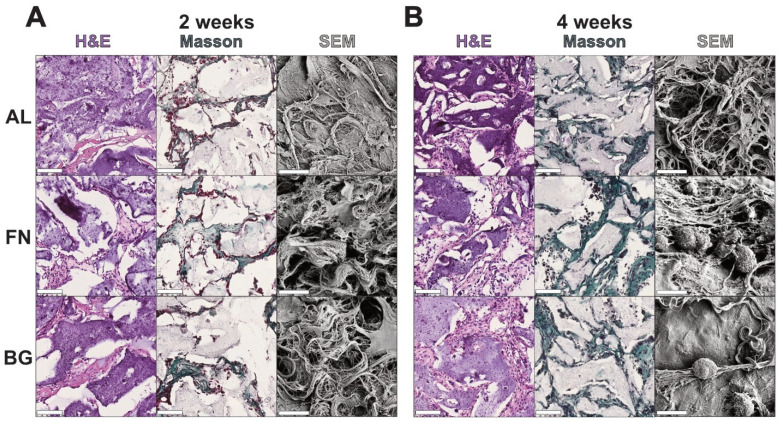
Histological Analysis of Scaffolds Post 2 (**A**) and 4-Weeks (**B**) Subcutaneous Implantation. H&E staining shows cell infiltration and tissue integration, with nuclei appearing dark purple and cytoplasm and extracellular matrix staining light pink. Masson’s Trichrome staining confirms cell nuclei (purple) and highlights collagen deposition in green, indicating tissue remodeling and scaffold integration. Insets display low-magnification overviews of entire scaffold sections. Scale bars represent 500 µm.

Histological and ultrastructural analyses were performed after 2 and 4 weeks of subcutaneous implantation to assess host cell infiltration and ECM deposition (Figure 5). H&E staining confirmed that all scaffolds supported host cell invasion, with an increase in cellular density over time. No major differences in cell distribution were observed among AL, FN, and BG scaffolds, indicating comparable tissue integration. Masson’s Trichrome staining revealed progressive collagen deposition, with FN scaffolds showing the earliest signs of fibrillar network formation. By 4 weeks, ECM accumulation increased across all scaffold types, though FN scaffolds exhibited a more structured matrix, while BG scaffolds showed a less organized collagen distribution.

SEM analysis further confirmed that all scaffolds remained structurally intact and exhibited increasing ECM deposition over time. While FN scaffolds displayed a denser fibrillar network, no strong material-dependent differences in scaffold remodeling were observed.

These findings demonstrate that all biomaterials tested were successfully invaded by host cells, supporting their basic biocompatibility. However, due to the lack of quantification and limited sample sizes, no conclusions can be drawn regarding differential scaffold performance; therefore, further studies are needed to comprehensively evaluate their long-term integration and functional potential.

The immunofluorescence (IF) staining (Figure S2) presents a comparative evaluation of cellular infiltration and vascularization within the four scaffold formulations (AL, FN, BG) after 4 weeks of subcutaneous implantation. Each scaffold is stained for DAPI (nuclear marker), Vimentin (Vim; mesenchymal cell marker), and CD31 (endothelial cell marker). Vimentin staining confirmed stromal cell infiltration across all scaffold types, with no significant differences in distribution or density between AL, FN, and BG scaffolds, suggesting a comparable ability to support mesenchymal cell invasion, confirming our previous results (Figure 5). CD31-positive regions were detected across all scaffold types (Figures S3 and S4), confirming the presence of endothelial cells. However, the signal was relatively weak and occasionally overlapped with scaffold architecture, raising the possibility of non-specific staining or autofluorescence. FN scaffolds appeared to have a slightly lower density of CD31+ cells compared to AL and BG, though this trend was not statistically confirmed. Given these limitations, the data are included for transparency but were not used to draw definitive conclusions regarding scaffold vascularization.

## 4. Discussion

Bone tissue engineering requires a translational strategy that combines in vitro and in vivo models to evaluate scaffold biocompatibility, integration, and resorption. While many studies rely on immortalized cell lines for initial screening, primary mesenchymal stromal cells (BM-MSCs) and adipose-derived stromal cells (ASCs) provide a more physiologically relevant model, capturing interpatient variability and more accurately reflecting clinical conditions [21]. As summarized in Table S2 (adapted from Refs. [11,12]), alginate/hydroxyapatite (AL) scaffolds functionalized with fibronectin (FN) or incorporating bioactive glass (BG) have previously been shown to enhance biocompatibility when tested with the human osteosarcoma cell line (MG-63). Due to their structural characteristics, these scaffolds remain promising candidates for supporting cellular activity and tissue integration in bone regeneration. While this study focused on the individual evaluation of AL, FN, and BG scaffolds previously validated by our group, a combination of fibronectin and BGMS10 may offer synergistic benefits and should be explored in future studies.

Here, we demonstrated that all three scaffolds supported adhesion and proliferation in immortalized M-SOD cells. In contrast, when using primary BM-MSCs and ASCs, adhesion remained strong, but proliferation was absent across all conditions. Osteoblastic differentiation was evident only in BM-MSCs, while ASCs showed limited osteogenic commitment. This aligns with known differences between these cell types: ASCs are generally more proliferative and adipogenic, whereas BM-MSCs are more prone to osteogenic and chondrogenic differentiation [22,23]. The limited osteogenic response of ASCs does not imply a complete lack of commitment but rather a reduced responsiveness under the same inductive conditions.

These findings confirm alginate’s biological inertness, which limits adhesion and proliferation due to insufficient biochemical cues [9]. Fibronectin improved cell adhesion, particularly with BM-MSCs, reinforcing the role of fibronectin in promoting integrin-mediated cell attachment and spreading [24,25]. In contrast, BG scaffolds did not significantly enhance early cell adhesion, suggesting that BGMS10’s benefits may require longer term culture to manifest [9,26,27]. After 21 days of osteogenic induction, BG scaffolds exhibited higher expression of late osteogenic markers, likely due to the release of magnesium and strontium ions known to enhance osteoblast activity and inhibit osteoclast resorption [28,29]. This is consistent with the BGMS10 ion release profile, which includes Si, Mg, and Sr (Supplementary Table S2). Despite these differences, both BM-MSCs and ASCs primarily formed rounded compact clusters on all scaffolds, indicating limited cell spreading and suboptimal surface bioactivity. Notably, scaffold structure remained stable over the 28 days culture period, supporting their mechanical suitability for long-term applications, an observation further confirmed in vivo.

While gene expression profiling revealed key insights into osteogenic progression, additional endpoint assays such as mineral deposition or immunostaining for late osteogenic markers were not feasible in the present setup. High hydroxyapatite content and poor adherence of cultured scaffolds to slides Prevented reliable histological or staining-based quantification. These limitations further underscore the value of our parallel in vivo model, which allowed assessment of early host infiltration and extracellular matrix deposition under physiological conditions. Future work should explore alternative strategies to complementary gene expression data.

To evaluate early host-material interactions under physiological conditions and avoid premature orthotopic implantation of poorly colonized scaffolds, we employed an acellular subcutaneous model. This approach also adheres to the 3R principle by limiting animal use. While orthotopic implantation in a critical-size bone defect model will ultimately be needed to assess regenerative capacity, subcutaneous models remain valuable for initial biocompatibility [30,31]. After four weeks, all scaffolds remained intact and were successfully infiltrated by host cells. However, no significant differences in cellular density or collagen deposition were observed between AL, FN, and BG groups, suggesting that surface modifications did not markedly influence host response at this early stage. Although ECM accumulation was evident, its organization and functional relevance remain to be elucidated. Previous studies have shown that structured ECM is essential for sustained remodeling and load-bearing capacity in bone graft [32].

Vascularization, as indicated by CD31 immunostaining, was minimal across all scaffolds, particularly in BG, where angiogenic effects might have been anticipated due to Mg^2+^ and Sr^2+^ release. [27] These ions are part of the BGMS10 profile and are known to contribute to angiogenesis and osteogenesis [12]. Assessing their bioavailability in vivo will be key to interpreting these outcomes. Future studies may incorporate pro-angiogenic cues (e.g., VEGF delivery) or adopt pre-vascularization strategies using co-culture or perfusion systems to improve early vascular integration [33,34].

Given these findings, orthotopic implantation models (e.g., mouse calvarial or femoral defect) will be necessary to assess long-term scaffold performance under mechanical and biological relevant conditions [35]. By combining in vitro analysis using primary human stromal cells with in vivo acellular implantation, we identified scaffold-specific limitations in cell proliferation and osteogenic differentiation that were not apparent using immortalized models, providing a more biologically relevant framework for early-stage scaffold evaluation and preclinical decision-making prior to orthotopic testing.

## 5. Conclusions

Although the scaffolds tested in this study demonstrated biocompatibility and supported host cell infiltration, the limited in vitro proliferation of primary stromal cells and lack of vascularization in vivo highlight key challenges for translation. Overcoming these biological limitations will be essential to advance these scaffolds toward clinical relevance. Once vascular integration is achieved, orthotopic implantation models will be required to fully evaluate regenerative efficacy under physiologically relevant conditions.

## Data Availability

The original contributions presented in the study are included in the article, further inquiries can be directed to the corresponding author.

## Supplementary Materials

The following supporting information can be downloaded at: https://www.mdpi.com/article/10.3390/jfb16100386/s1, Figure S1: Scaffold Manufacturing Process Using 3D-Printed PLA Slicer; Figure S2: Light Microscope Images Showing Cells Adhesion and Distribution on AL (a–c), FN (d–f), and BG (g–i) Scaffolds on Day 1; Figure S3: Vascularization of scaffolds post 4 weeks implantation; Figure S4: Quantification of CD31+ cells across scaffold types; Table S1. Details and catalogs numbers of reagents used in this study; Table S2: Physicochemical and mechanical properties of scaffold formulations.
