# Supplementary material for: Fibronectin- and Bioactive Glass-Modified Alginate Scaffolds Support Limited Primary Cell Proliferation In Vitro yet Demonstrate Effective Host Integration In Vivo
Source: J Funct Biomater. 2025 Oct 15;16(10):386. doi: 10.3390/jfb16100386 (PMC12565557; doi:10.3390/jfb16100386)
Supplement: Supplementary file 1 [file jfb-16-00386-s001.zip › jfb-3870861-supplementary.pdf]

Fibronectin- and Bioactive Glass-Modified Alginate Scaffolds Support Limited Primary Cell Proliferation In Vitro yet Demonstrate Effective Host Integration In Vivo

SUPPLEMENTARY MATERIAL

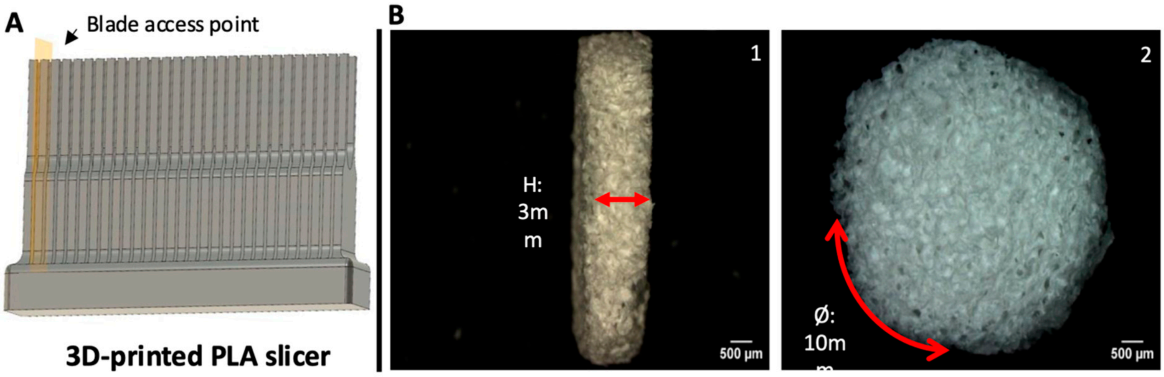

Figure S1. Scaffold Manufacturing Process Using 3D-Printed PLA Slicer. Panel A shows a 3D-printed PLA slicer used to cut cylindrical scaffold slices precisely. The design includes a blade access point allowing precise sectioning of the scaffold material. The slicer ensures uniform cuts for scaffold consistency. Panel B-1 presents a side view of the scaffold slice with a height (H) of 3 mm, while Panel B-2 shows a top view of the same slice with a diameter (Ø) of 10 mm. Scale bars represent 500 µm. Both images demonstrate the uniformity of scaffold dimensions, ensuring reproducibility in subsequent experiments.

**Method Section**	**Reagent**	**Company**	**Catalog Number**
**Culture Media**	α-Modified Eagle's Medium (α-MEM)	Gibco, Thermo Fisher Scientific	22571
	Fetal Bovine Serum (FBS)	Gibco, Thermo Fisher Scientific	12491
	GlutaMAX	Gibco, Thermo Fisher Scientific	35050

	HEPES buffer solution	Gibco, Thermo Fisher Scientific	15630
	Sodium pyruvate	Gibco, Thermo Fisher Scientific	11360
	Penicillin-Streptomycin (PENSTREP)	Gibco, Thermo Fisher Scientific	15140
	Fibroblast Growth Factor-2 (FGF2)	R&D Systems, Bio-Techne	3718-FB
	Trypsin-EDTA	Gibco, Thermo Fisher Scientific	25300
**Materials and Scaffold Manufacturing**	Sodium alginate	Sigma-Aldrich, Merck KGaA	Provided by FMC Biopolymers (Dramen, Norway)
	Hydroxyapatite (HAp)	Sigma-Aldrich, Merck KGaA	21223
	Bioactive glass (BGMS10)	Sigma-Aldrich, Merck KGaA	Raw material for biaoctive glass preparation were purchased from Carlo Erba (Milan, Italy)
	Glucono-delta-lactone (GDL)	Sigma-Aldrich, Merck KGaA	G4750
	Fibronectin	Sigma-Aldrich, Merck KGaA	F1141
**Adhesion and Proliferation**	MTT Solution	Sigma-Aldrich, Merck KGaA	M6494
	CellTiter-Glo Assay	Promega	G9682
**Scanning Electron Microscopy (SEM)**	Sodium cacodylate buffer	Thermo Fisher Scientific	15404619
	Glutaraldehyde	Life Technologies	A17876.AE
**RNA Extraction and qRT-PCR**	TRI Reagent Solution	Sigma-Aldrich, Merck KGaA	T9424
	Qiagen Miniprep Kit	Qiagen	27104
	SuperScript III Reverse Transcriptase	Invitrogen, Thermo Fisher Scientific	18080093

	Runx2	Thermo Fisher Scientific	Hs00231692_m1
	Osteocalcin (Bglap/Ocn)	Thermo Fisher Scientific	Hs01587814_g1
	Ki-67	Thermo Fisher Scientific	Hs01032443_m1
	Osteopontin (Opn)	Thermo Fisher Scientific	Hs00959010_m1
	Bone Sialoprotein (Ibsp)	Thermo Fisher Scientific	Hs00173720_m1
	Collagen type I (Col1a1)	Thermo Fisher Scientific	Hs00164004_m1
	Alkaline Phosphatase (Alpl)	Thermo Fisher Scientific	Hs01029144_m1
	GAPDH (Housekeeping)	Thermo Fisher Scientific	Hs02758991_g1
**Whole Mount Immunostaining and Confocal Imaging**	FITC-Phalloidin	Sigma-Aldrich, Merck KGaA	P5282
	Rat anti-human Ki-67 primary antibody	Sigma-Aldrich, Merck KGaA	14-5698-82
**Immunofluorescence Staining**	Ultraclear	J.T. Baker	3905.5000PE
	Citrate Buffer (pH 6)	Protaqs	400300692
	Rat anti-mouse CD31	Abcam	ab32457
	Rabbit anti-human Vimentin	Abcam	ab92547
	AlexaFluor 647 anti-Rat	Invitrogen, Thermo Fisher Scientific	A21247
	AlexaFluor 546 anti-Rabbit	Invitrogen, Thermo Fisher Scientific	A11035
	DAPI	Invitrogen, Thermo Fisher Scientific	564907
**Histological Processing and Staining**	Hematoxylin		#NotProvided
	Eosin		#NotProvided
	Masson's Trichrome Staining Kit		#361350

Table S1. Details and catalogs numbers of reagents used in this study.

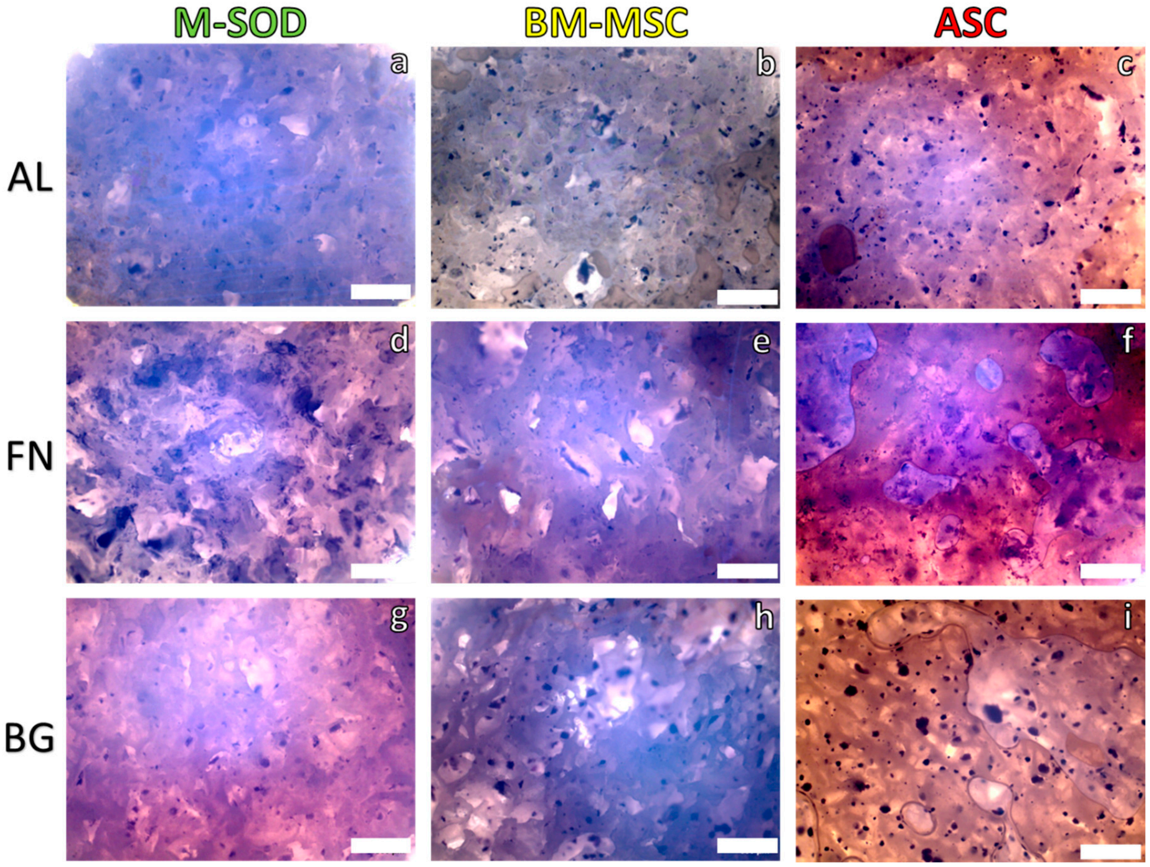

Figure S2. Light Microscope Images Showing Cells Adhesion and Distribution on AL (a-c), FN (d-f), and BG (g-i) Scaffolds on Day 1. Dark spots indicate cell clusters metabolizing the MTT dye, reflecting cell viability. Scale bar: 500 µm.

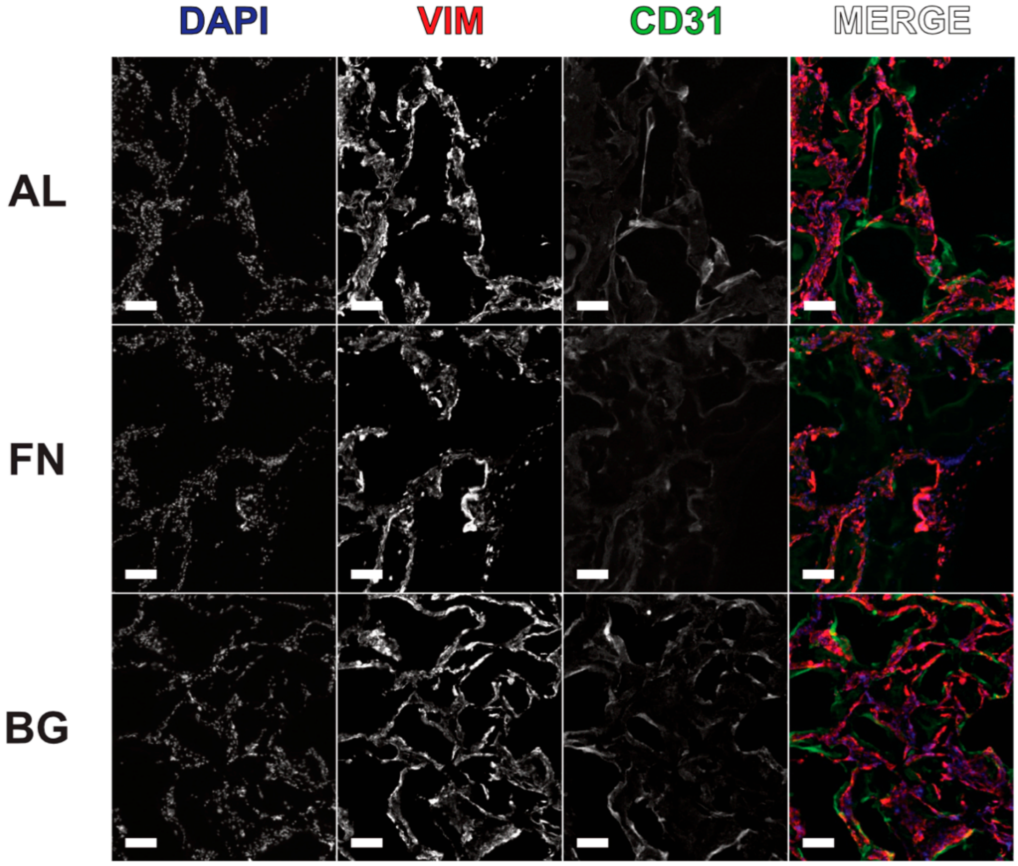

Figure S3. Vascularization of scaffolds post 4 weeks implantation. The limited CD31 signal suggests minimal endothelial presence and thus low vascularization. DAPI staining confirms the widespread presence of nuclei, indicating active cellular colonization. Individual channels are shown in greyscale to enhance contrast; colour coding for each marker is indicated in the panel headers (DAPI, blue; VIM, red; CD31, green). The MERGE panel combines these colours. Scale bars represent 100 µm.

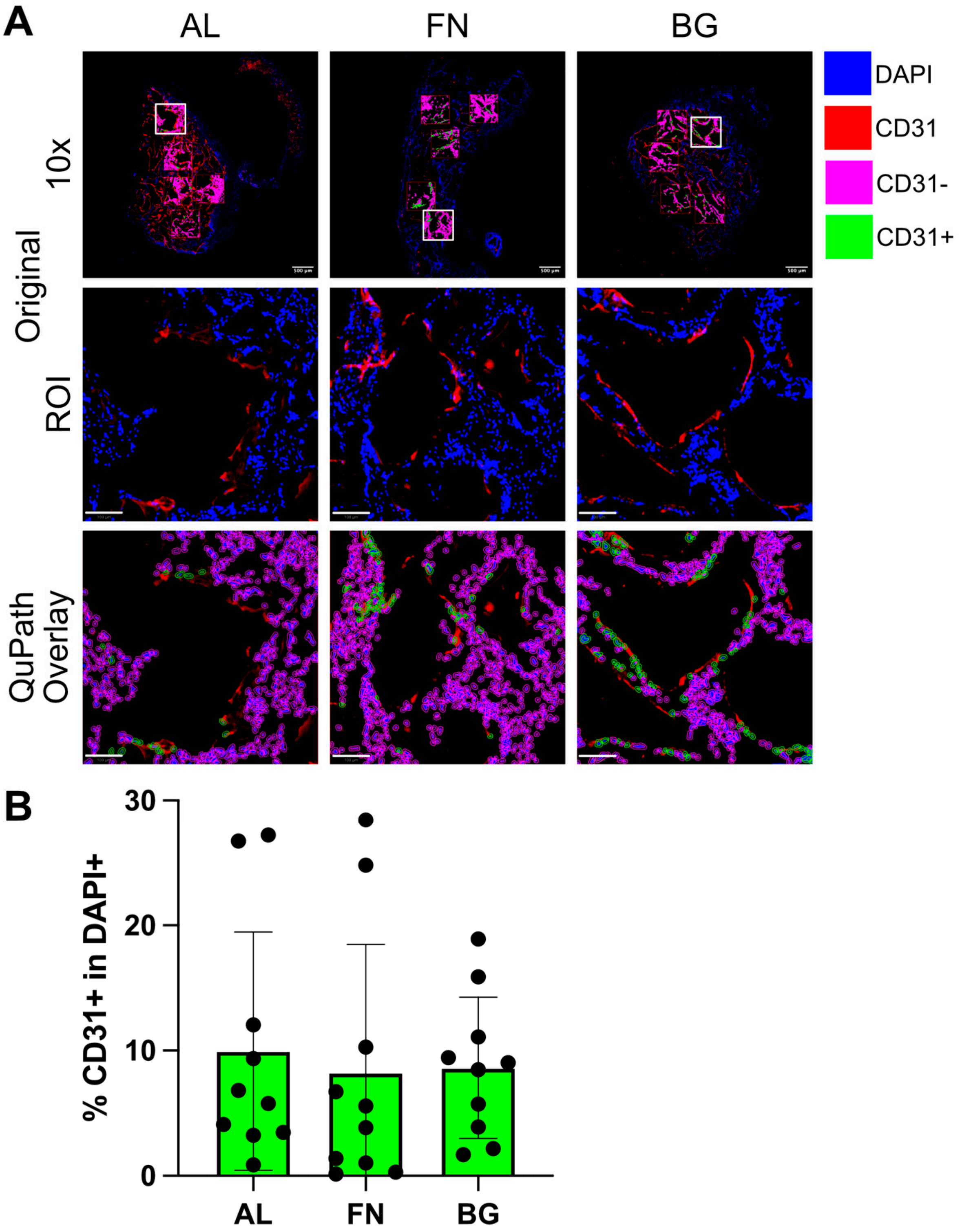

Figure S4. Quantification of CD31+ cells across scaffold types. (A) Representative immunofluorescence images of AL, FN, and BG scaffolds after 4 weeks in vivo (scale bar: 500µm), stained for DAPI (blue), CD31 (red), and QuPath $CD31^{+}$ segmented overlay (green).

$CD31^-$ $DAPI^+$ nuclei are shown in magenta (scale bar: 100 µm). (B) Quantification of $CD31^+$ cells as a percentage of total $DAPI^+$ cells using QuPath analysis. Data are shown as mean ± SD. No statistically significant differences were observed between groups.

Property	AL (Alg/HAp)	BG (Alg/HAp + BGMS10)	FN (Alg/HAp + Fibronectin)
**Porosity (%)**	>88%	80.2 ± 1.1% (0.3% w/v BGMS10) 70.2 ± 0.6% (0.6% w/v BGMS10)	>88%
**Pore size (µm)**	100–300	100–300 (slightly reduced interconnectivity at higher BG load)	100–300
**Surface morphology**	Rough pore walls due to HAp granules	Smoother areas through cavities due to BG dissolution and apatite precipitation	Rough pore walls covered by thin FN coating
**Alginate content (% w/v)**	2%	2%	2%
**HAp content (% w/v)**	3%	3%	3%
**HAp particle size**	150 nm	150 nm	150 nm
**BGMS10 content**	–	0.3% or 0.6% (w/v)	–
**BGMS10 particle size**	–	<63 µm	–
**FN coating amount**	–	–	10 µg/mL adsorption
**Ion release profile**	Ca^{2+} and PO_4^{3-} release during gelation (~5% HAp dissolution)	Release of Si, Ca, Na, P, Mg, Sr over time in PBS; Mg decreases markedly; Si and P decrease slightly due to apatite formation	Not applicable
**pH effect during fabrication**	GDL lowers pH to facilitate Ca^{2+} release and gelation	Same as AL; BGMS10 stable under gelation conditions	Same as AL
**Dry compressive modulus (MPa)**	~6.3	~6.3 (BG6-sc)	Assumed similar to AL
**Dry ultimate compressive strength (MPa)**	~0.29	~0.29 (BG6-sc)	Assumed similar to AL
**Hydrated compressive modulus (kPa)**	~83	~83 (BG6-sc)	Assumed similar to AL
**Weight loss in water (%)**	14% after 31 days	Slightly reduced compared to AL	Not reported separately
**Swelling ratio**	~1400% (w/w) in 5 min	Reduced vs AL due to lower porosity	Similar to AL
**Key biological implication**	High porosity, HAp ion release supports osteoblast proliferation	BG ion release (Si, Mg, Sr) promotes osteogenesis, angiogenesis	FN enhances initial cell adhesion and spreading
**Structure (SEM)**	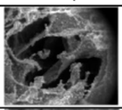	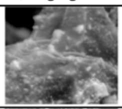	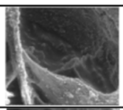
**Cells Morphology (MG-63)**	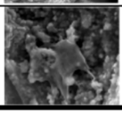	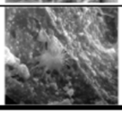	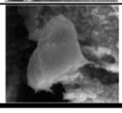

Table S2. Physicochemical and mechanical properties of scaffold formulations

Values derived from previously published characterizations using identical fabrication protocols and raw materials. . Adapted from [11, 12].
